# Measuring Temperature Induced Phase Change Kinetics in Subcutaneous Adipose Tissues Using Near Infrared Spectroscopy, MR Imaging and Spectroscopy and OCT

**DOI:** 10.1038/s41598-017-18145-9

**Published:** 2017-12-19

**Authors:** Amir Y. Sajjadi, Dieter Manstein, Stefan A. Carp

**Affiliations:** 1 Athinoula A. Martinos Center for Biomedical Imaging, Department of Radiology, Harvard Medical School, Massachusetts General Hospital, Charlestown, MA 02129 USA; 2 Cutaneous Biology Research Center, Department of Dermatology, Harvard Medical School, Massachusetts General Hospital, Charlestown, MA 02129 USA

## Abstract

Monitoring phase transition in adipose tissue and formation of lipid crystals is important in Cryo-procedures such as Selective Cryolipolysis (SC). We exploited a Near-Infrared Spectroscopy (NIRS) method to monitor the onset of fat phase transition (freezing/melting) in human abdominal adipose tissue. The changes in optical scattering were compared to Differential Scanning Calorimetry (DSC) measurements as the gold standard method for measuring phase transition. For some samples, concurrent *in vitro* measurements of optical scattering using NIRS and the MR signal parameters (T_2_*) as well as spectral parameters using MR Spectroscopy were performed in a 3 T MR scanner during a cooling/heating cycle. To further investigate phase-transition in adipose tissue in microscopic level, an identical cooling/heating procedure was replicated on a small piece of fat harvested from the same tissue while being imaged under Optical Coherence Tomography (OCT). For all methods, their relationship with temperature shows inflexions in a narrow range, characteristic of lipid phase transition. In particular, the good agreement between DSC and Optical measurements suggests that such NIRS methods can be used to improve dosimetry and to minimize variations of clinical outcome for cryo-procedures.

## Introduction

Selective Cryolipolysis (SC) is a non-invasive medical treatment to destroy fat cells by controlled use of cooling^1,2^. Since SC was introduced, its safety and efficacy has been shown in several studies^3–7^ and is spreading quickly around the world. In principle, controlled cooling of fat tissue causes non-invasive localized reduction of the fat deposits. In SC, the machine generates vacuum to encompass the adipose tissue and reduce the local blood pressure^1^, while cooling the adipose tissue to lower the intra-adiposity temperature. This is thought to cause cellular changes to an extent that generates therapeutic results without damaging other structures^8^. Formation of lipid crystals (fat freezing) may play an essential role in inducing localized destruction of fat deposits. Currently SC is performed with area- and applicator-specific preset treatment settings (time, cooling rate, and preset temperature). Although the clinical studies have demonstrated the efficacy of cryolipolysis for subcutaneous fat removal, the exact mechanism of action for cryolipolysis is not yet completely understood. Studies have shown that multiple additional pre/post treatment options such as massage can enhance the efficacy of the outcome^9,10^. This procedure is proved to be effective for removal of subcutaneous fat^11^, however it is conceivable that real time monitoring of onset and distribution of fat freezing can improve the dosimetry of the treatment and further minimize any variation of treatment efficacy. Understanding the onset of changes in the morphology of the fat tissue during cooling and the kinetics of fat phase change at different temperatures can help in investigating the mechanism of fat cell removal. It was proposed that phase changes could be lipid crystallization or lipid-to-gel phase transition. Currently, there are no non-invasive techniques to monitor these changes even *in vitro*. The composition of fatty acids whether saturated or non-saturated, undergoes crystallization at different temperature^4–6^. It has also been shown that the exposure time affect the crystallization process for example, samples exposed to 8 °C for 25 min have larger crystal size compared to the samples exposed for 10 min^12^. The variation in amount of unsaturated fat between patients and the fact that mono and poly saturated fatty acids have different liquid-to-gel transition points^13,14^ suggest that each patient experiences different crystallization kinetics during cryo-procedures. Thus, monitoring the kinetic of these changes in each individual patient can help enhancing the treatment outcome.

In this work, we investigate the onset and evolution of phase change in subcutaneous fatty tissue during the cooling/heating using Near-Infrared Spectroscopy (NIRS). Changes in optical properties (in particular the scattering µ_s_′ coefficient), which can be measured using NIRS, can reveal changes in the morphology of the tissue during the cooling procedure. We compare the Optical Scattering (OS) measurements to Differential Scanning Calorimetry (DSC), the gold standard for monitoring phase transitions. For some samples we also performed Magnetic Resonance Imaging (MRI)/Spectroscopy (MRS) measurements as well as Optical Coherence Tomography.

## Materials and Methods

### Tissue Samples

All measurements were conducted under a protocol approved by the Massachusetts General Hospital Institutional Review Board and all methods were performed in accordance with the relevant guidelines and regulations. All patients involved in the study signed an informed consent form including consent to participate in the study and to publish the anonymized data. Two human skin specimens with a thick subcutaneous fatty layer were harvested from the byproducts of abdominoplasty procedures (fat layer thickness 1.5–3 cm). The first was further divided in 4 pieces, while the second, smaller specimen was divided into two halves. The tissues were kept in a 4 °C refrigerator or a −20 °C freezer (if longer storage was needed) prior to being measured. Before the measurement session, samples were rewarmed to body-like temperature (37–40 °C) for at least 30 minutes, and were wrapped in thin transparent plastic foil for easier manipulation.

### Cooling/Heating Process

We utilized two water/antifreeze operated heaters/chillers to deliver heating/cooling inputs: a Julabo FL-601 (Julabo USA, Inc.) and a Stryker Gaymar TP700 (Stryker, Inc.). The higher capacity Julabo unit was used as the cold source (set to −10 °C), while the Stryker was used as the warm source (set to 42 °C). The switching was accomplished using a system of valves that allowed both devices to circulate continuously while switching one of them at a time into the cooling/heating pad circuit. This was done to accelerate cycling time by avoiding the need for the large thermal mass of the operating fluid to vary between the low and the high temperature setting.

**Figure 1:**
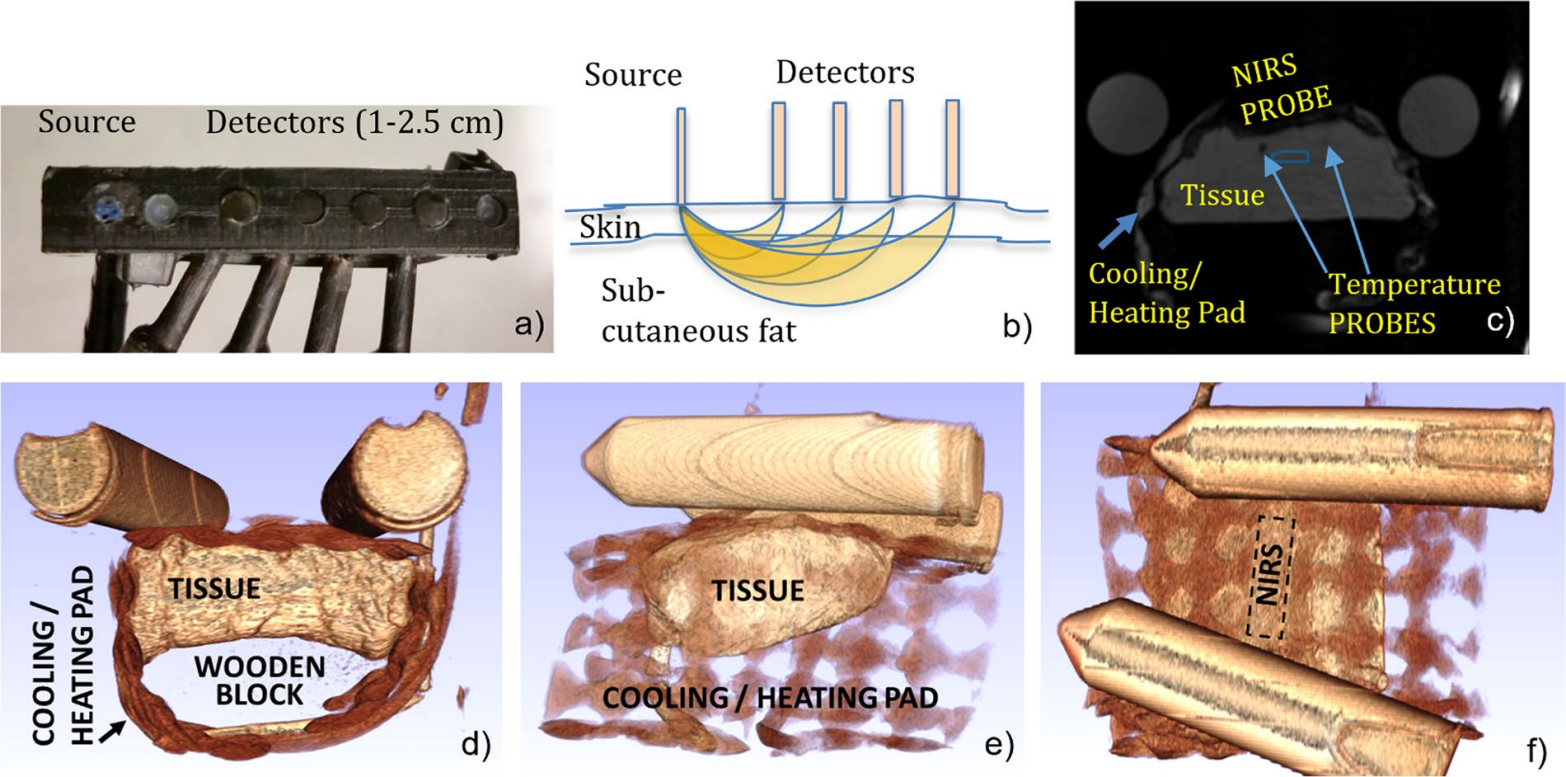
(**a**) Photograph of the NIRS probe. Three 400 micron fibers deliver 690, 782 and 830 nm light at the source location, while 4 2.5 mm fiber bundles collect diffusely reflected light at distances of 1, 1.5, 2 and 2.5 cm; (**b**) Schematic expected sensitivity profiles for the NIRS measurements and their spatial relationship to the skin and subcutaneous fat layers. (**c**) T1-weighted MR image slice (centered under NIRS probe) showing the location of the sample, cooling/heating pad, temperature probes and NIRS probe for simultaneous NIRS-MR measurements. The blue region shows the location of MR Spectroscopy voxel. (**d**) front, (**e**) side and (**f**) top view of 3D rendering of a T1-weighted MR scan showing the tissue sample, the fluid network inside the cooling pad, and the outline of the NIRS probe placement (the tube structures on top are oil filled vials used to track the scanner field drift).

Cooling/heating was applied using a flexible rectangular pad wrapped around the sample (Cold Rush, Ossur Inc.). The thermal pad encompasses the tissue such that it cools/heat the surface, and in order to guarantee the exchange of heat happens only from the surface of the skin, a wooden block was placed underneath the tissue, between the fat and the bottom of the thermal pad. The NIRS probe (below) is positioned on the skin through an opening cut in the center of the pad, and is used to measure the optical properties of the adipose tissue. The internal temperature inside the sample was monitored using two 1.15 mm diameter T1 fiber optic temperature probes attached to a Neoptix Reflex head unit (Qualitrol Inc.) that offered logging over RS-232. The probes were inserted through the face of the sample not covered by the thermal pad and pushed in such that their tips reached the area under the NIRS probe at depths of ~5 mm and ~8 mm (matching expected NIRS depth sensitivity profile). Figure 1c–f) show an MRI image and three views from a 3D rendering of the experimental set-up from an MRI structural scan. The arrangement was similar for NIRS only experiments.

Each sample was first heated to body-like temperatures (37–40 °C) as described above using a hot plate. Then water circulation through the cooling/heating pad was established using the Stryker device set to 42 °C. After ~10 minutes of baseline, the circulation was switched to the Julabo device set to −10 °C until the sample internal temperature (as reported by the fiber optic thermometer) approached 0 °C. At that point, the circulation was switched back to Stryker device for warming, and measurements continued until the sample internal temperature reached at least 35 °C.

### Near Infrared Spectroscopy

We used an ISS Imagent Frequency Domain Spectrometer (ISS Inc.) operating at a modulation frequency of 110 MHz. The instrument offers eight time-shared laser diodes per source bank at wavelengths between 635 and 830 nm, as well as four photomultiplier tube detectors. A custom made probe (see Fig. 1a) used 3 co-localized 400 micron optical fibers to deliver 690, 782 and 830 nm light at one location, and provided 4 2.5 mm fiber bundles to collect the diffusely reflected light at distance of 1, 1.5, 2 and 2.5 cm from the source location, respectively. The distance range was chosen to provide sensitivity to the sub-cutaneous fat layer (expected depth sensitivity profiles are shown in Fig. 1b). The probe was inserted through a cut in the center of the cooling pad and was in direct contact with the skin side of the sample. Tissue optical properties (absorption (µ_a_) and scattering (µ_s_′) coefficients) are derived from measurements at the multiple source-detector separations using the multi-distance approach described in^15^, in conjunction with calibration measurements taken using a reference block.

### Magnetic Resonance Imaging and Spectroscopy

Scans were performed using a Siemens Prisma 3 T MRI scanner and a 64 channel head coil. The sample was wrapped in the cooling/heating pad, the NIRS probe and fiber optic temperature sensors were inserted, and the assembly was placed on a custom-built low thermal conductance platform that ensured the tissue was close to the center of the MR coil. Several foam blocks were used to immobilize the sample assembly. Both optical fibers (NIRS and temperature sensing) as well as the cooling/heating hoses were routed through access ports and the instruments/temperature cycling devices were placed in the MR scanner instrumentation room.

For each sample we acquired a 3D-FLASH T1-weighted structural scan to visualize the contact between the fluid pad and the sample, as well as proper positioning of the NIRS and temperature probes (see Fig. 1c). Then, a slice location was selected in the area approximately under the NIRS probe, and was imaged repeatedly using a 2D T1-weighted multi-echo gradient echo sequence (TR/TE/FA = 150 ms/2.9–30 ms/70°). Single voxel spectroscopy (SVS) data was also acquired in an interleaved fashion with the 2D GRE images, using a PRESS sequence with a TE of 30 ms. The SVS voxel was placed near the fiber optic temperature sensors, approximately in the area probed by the NIRS measurement. Both MR sequences were repeated for as long as needed to complete the cooling/heating cycle.

The multi-echo data was then fit to an exponential decay to obtain the T2* relaxation time. Its value was computed over time in a region-of-interest defined to approximately coincide with the area probed by NIRS. The MR spectroscopy spectra were fit using Lorentzian curves to obtain the peak location, height, full-width at half-max and peak area. A typical MR spectrum of fatty tissue measured by clinical field strengths has six distinct fat peaks although the peaks 1 and 2 cannot be clearly distinguished from water^16^. We focused our analysis on the methylene peak at ~1.3 ppm.

### Optical Coherence Tomography

To investigate the microscopic behavior of fatty tissue during cooling/heating, small (~1.5 mm^3^) pieces of the subcutaneous fat were cut off the tissue samples prior to bulk temperature cycling. These samples were separately imaged using a Thorlabs Telesto 1300 nm spectral domain OCT system^17^. The sample was positioned on a thermoelectrically cooled/heated platform and imaged as the temperature was varied according to the recorded temperature time-course from the bulk tissue measurements. The temperature was monitored using an Omega thin-wire thermocouple. Averaged B-scan images were saved every second for the duration of the temperature playback. OCT frames were then “flattened” by lateral averaging into a single intensity vs depth decay profile, and fit to a single exponential decay model. The surface signal intensity was extrapolated from the model at zero depth, while the exponential attenuation coefficient was used as the effective optical attenuation of the sample.

### Differential Scanning Calorimetry

Several small pieces were also harvested from each tissue sample before the bulk tissue experiments, and were sent on dry ice to an outside service (Netzsch Instruments, Burlington, Mass.) for characterization using Differential Scanning Calorimetry. Measurements were performed using a Netzsch DSC 214 Polyma device. Three cooling/heating cycles were performed from 45 °C to −20 °C and back to 45 °C at 5 degrees per minute, using ~10 mg of fatty tissue sample while recording the energy flow to/from the sample.

## Results

**Figure 2:**
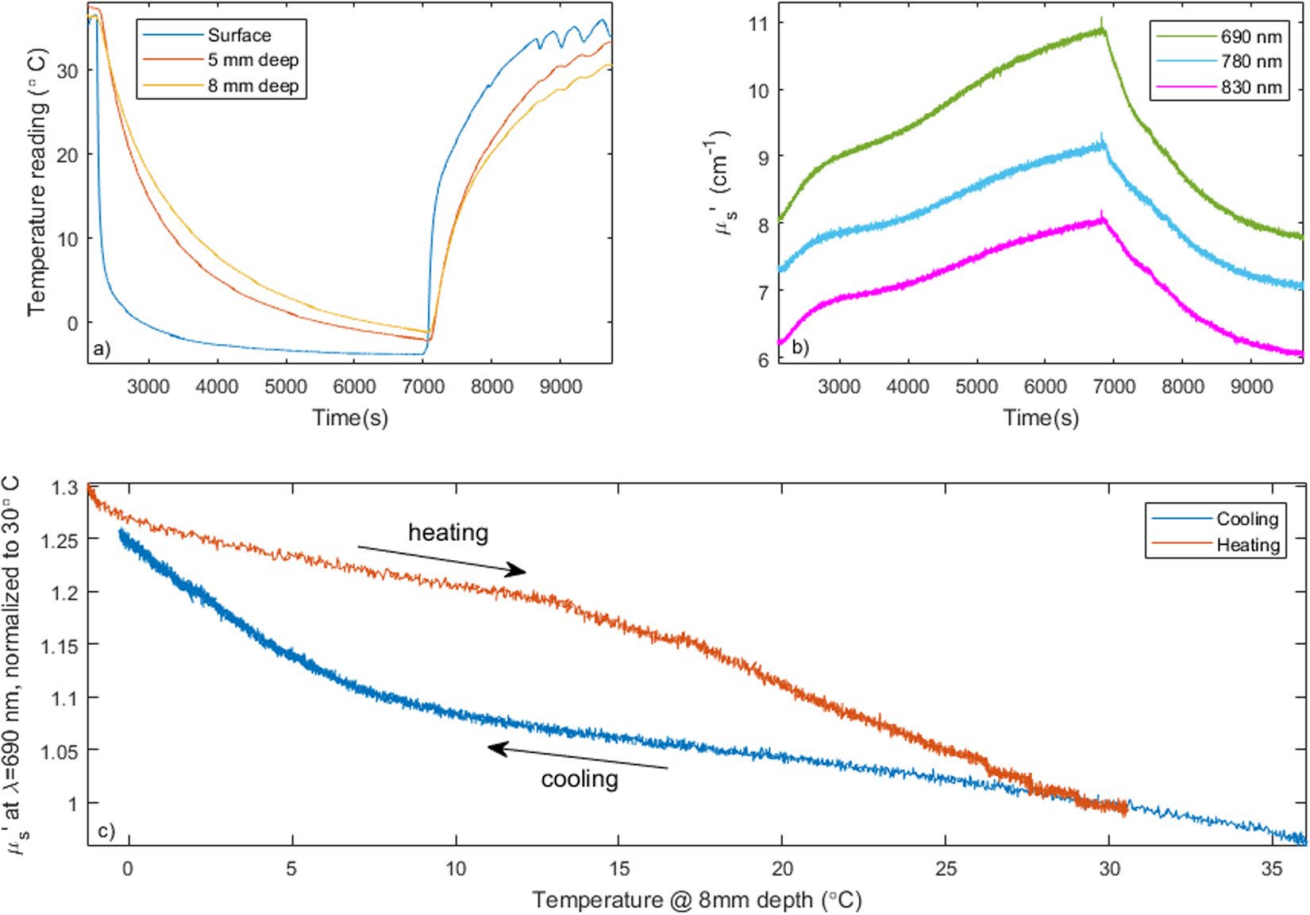
(**a**) Example temperature timeline during a cooling/heating cycle. The blue line shows the temperature of the cooling/heating pad, while the red and orange lines reflect the internal temperature of the sample at different depths. (**b**) Scattering measurement at 690, 780 and 830 nm during cooling. (**c**) Optical scattering vs. temperature. The blue line corresponds to the cooling cycle, while the red corresponds to the heating cycle.

Figure 2 shows an example of scattering measurements during a cooling/heating cycle. Panel a) displays the temperature at the surface (a) and at two different depths in the tissue. The pad (surface) temperature (blue) was initially 36 degrees, quickly brought down to below 0, then heated up to 35 degrees once the tissue temperature reached approximately 0 degrees. The two internal thermal probes (red, orange) show similar temperature profiles, reflecting the delayed propagation of the cooling front inside the tissue. The 1st probe was inserted ~2 mm deeper than the second and thus the cooling was slightly slower and less deep. Scattering coefficient values over time for the three different wavelengths are shown in Fig. 2(b). The optical scattering increases with decreasing temperature and decreases with increasing temperature. The similarity in the behavior at the three wavelengths suggests that only one wavelength would be sufficient for effective tissue monitoring. Figure 2(c) displays the direct relationship of scattering with temperature at 690 nm during cooling (blue line) and heating (red line). During the cooling trace (blue) the optical scattering progressively increases, but the increase substantially accelerates once the sample goes under ~10 degrees C. During the heating cycle (red), the scattering progressively decreases, but the decrease sharply accelerates once the sample heats beyond approximately 14 degrees C.

**Figure 3:**
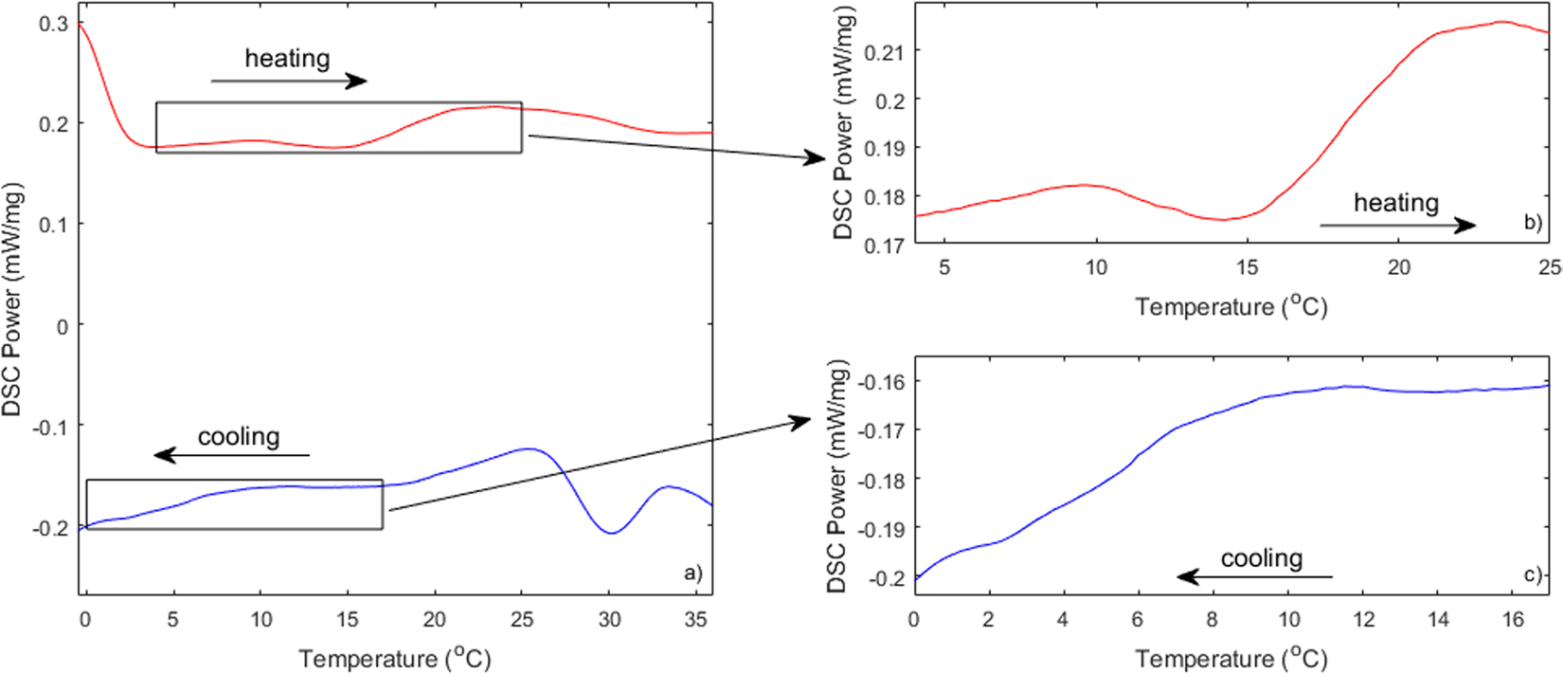
Differential Scanning Calorimetry traces for tissue sample in Fig. 2. (**a**) Complete heating and cooling data; (**b**) Heating detail in the 4 to 25 degree temperature range; (**c**) Cooling detail in the 0 to 16.5 degree temperature range.

Differential Scanning Calorimetry (DSC) is the gold standard method for measuring phase transitions in the tissue. Figure 3 shows differential scanning calorimetry data for a small portion of the tissue sample reported in Fig. 2, harvested from the subcutaneous fat layer. While several features are observed over the entire temperature range probed, an increase in the apparent heat capacity is seen below ~10 degrees during the cooling (see inset in panel b) and above 15 degrees during the heating, indicative of a potential phase transition in the fatty tissue.

**Figure 4:**
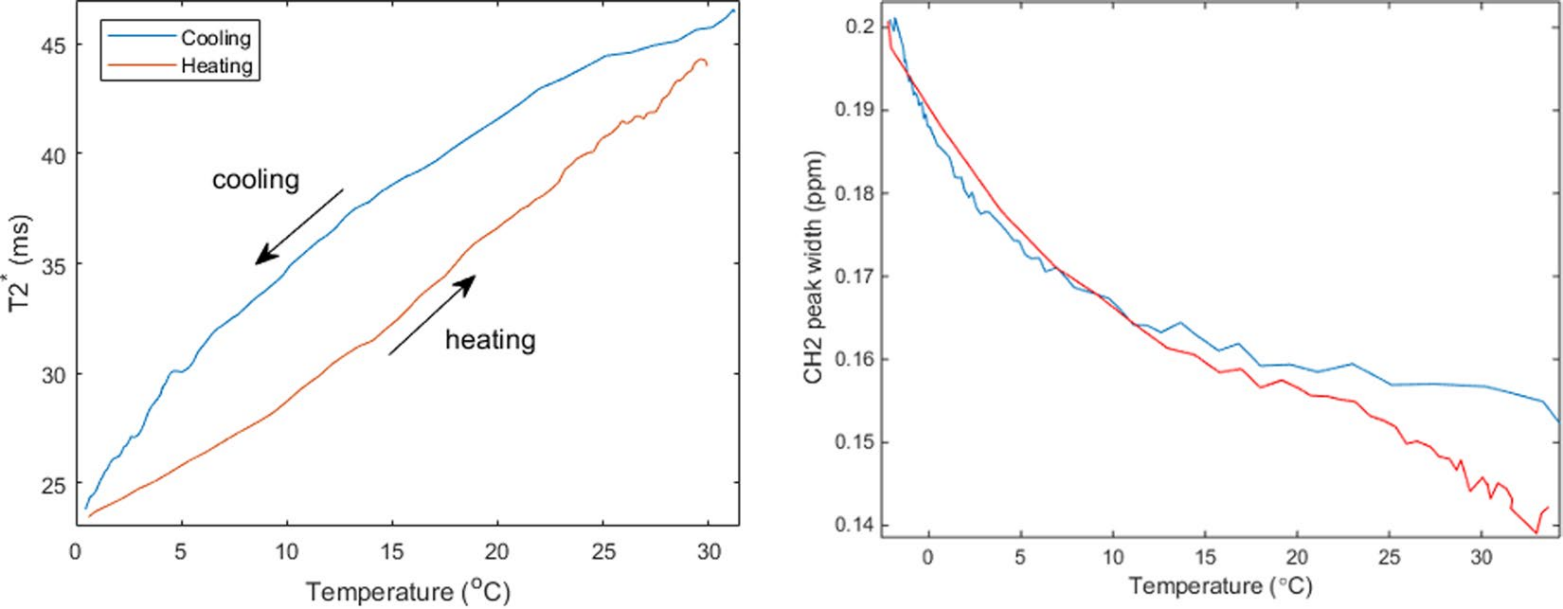
(**a**) MR T2* dependence on temperature during cooling (blue) and heating (red). (**b**) Methylene (-CH2-) peak width in ppm vs temperature for the tissue sample in Fig. 2 during cooling (blue) and heating (red) periods.

Figure 4 shows MR based data acquired on the same sample. In both cases the temperature reading from the 8 mm deep temperature probe was used as the x-axis. Panel a) displays the changes in the T2* relaxation time with temperature – a slow decrease is seen during cooling that accelerates once the sample passes ~7–9 degrees; during heating the T2* progressively increases, and the increase appears to slightly accelerate once the tissue is heated beyond ~13 degrees. The fat spectrum acquired using MR single voxel spectroscopy shows broadening of multiple fat peaks. Panel b) displays the width of the -CH_2_- (methylene) peak obtained by fitting the MR spectroscopy data. The evolution during heating and cooling is nearly identical, and there is no distinctive inflexion in the temperature relationship.

**Figure 5:**
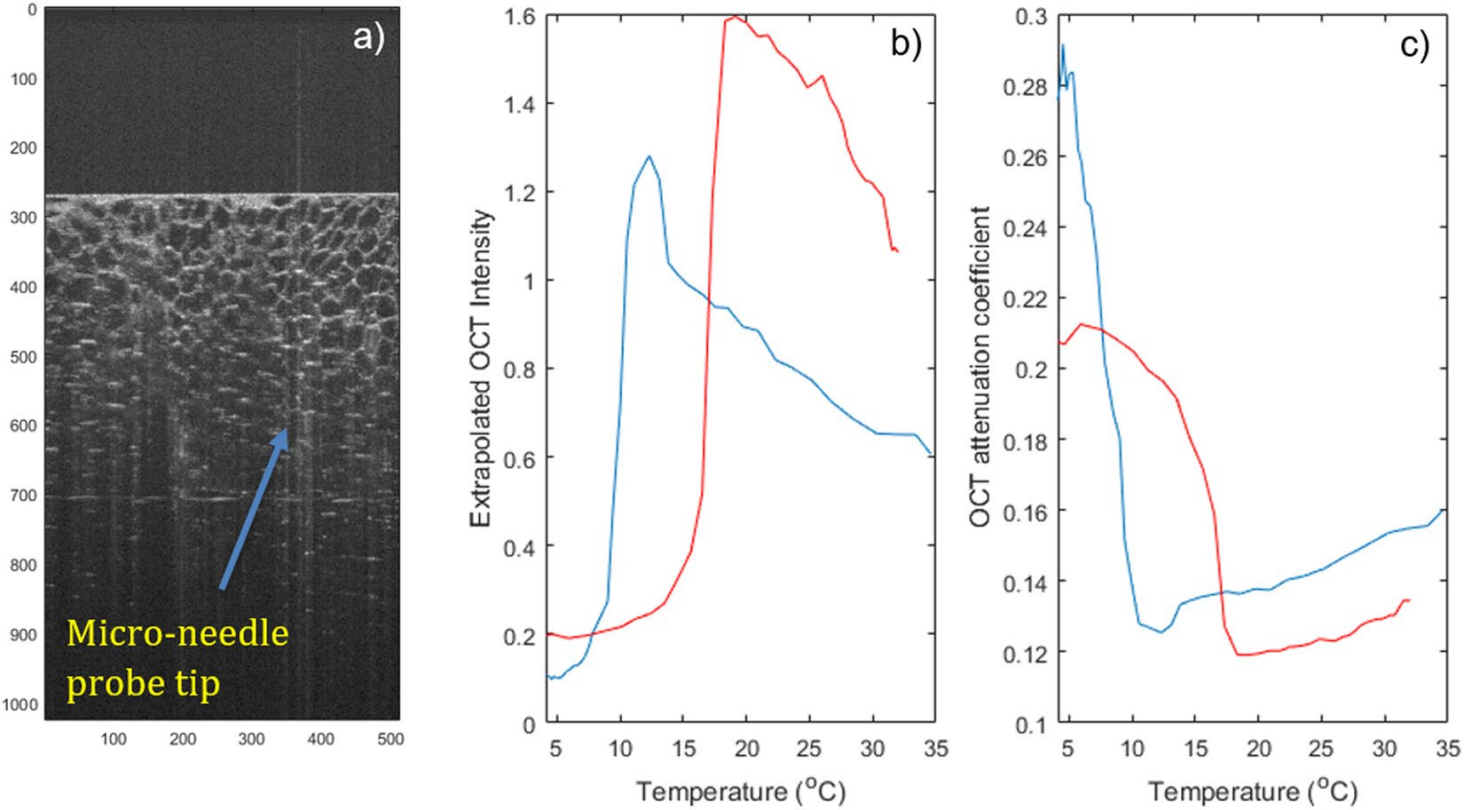
Example OCT data. (**a**) B-Scan showing cellular structure; (**b**) Extrapolated signal intensity averaged over the horizontal dimension of the image; (**c**) OCT attenuation coefficient averaged over the horizontal dimension of the image. The location of the tip of the micro-needle probe used to monitor temperature is indicated by an arrow.

We further utilized Optical Coherence Tomography (OCT) to study the microscale phase transition. Figure 5 shows an OCT measurement conducted on a small (1 mm) piece of fat from the same sample reported above (Fig. 2) which was harvested prior to the bulk tissue measurements and was thermally cycled using the temperature profile recorded during the measurements reported in Fig. 2 above truncated to 4 degrees C. The first panel in Fig. 5 shows an example B-Scan image revealing the cellular structure of the sample. The image was then averaged horizontally at each frame and thus compressed into a single decay profile. This profile was fit with an exponential model, and panel b) shows the extrapolated surface signal intensity from this fit, while c) shows the effective attenuation coefficient of the OCT signal intensity vs. depth. While more comprehensive models that take into account beam parameters have been used to process OCT data^18^, we believe the simple single exponential attenuation model used here is sufficient to capture the substantial change in sample optical properties observed during the phase transition. As the sample cools (blue trace) there is a sudden drop in signal intensity as the temperature decrease below ~10 degrees accompanied by a similarly sharp increase in attenuation. The behavior reverses as the sample is reheated past ~15–16 degrees with a sharp increase in signal and an associated decrease in attenuation (red trace).

**Figure 6:**
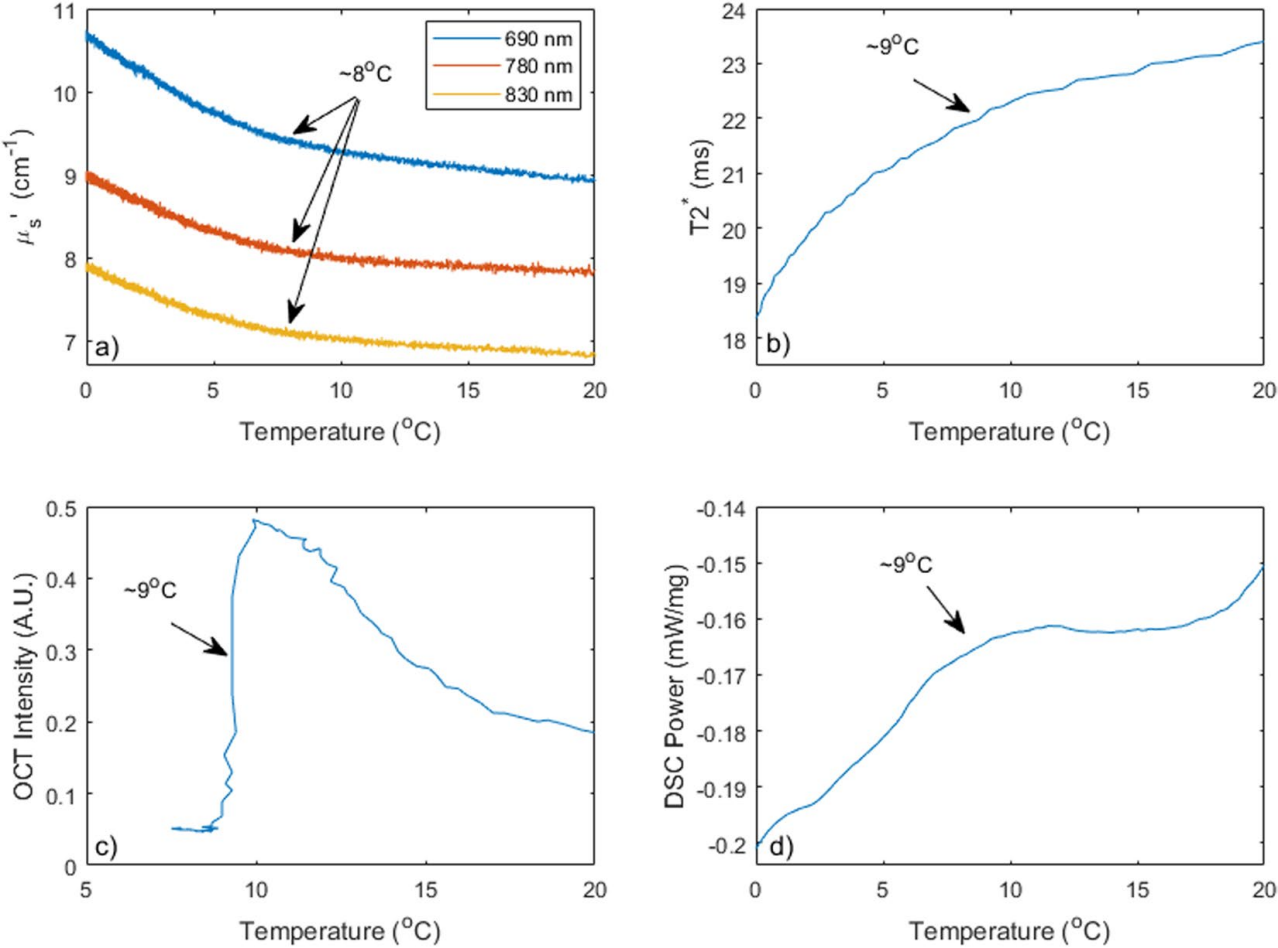
Comparing optical scattering (**a**), MR T2* (**b**), OCT (**c**) and DSC (**d**) on a sample of abdominal human skin with thick subcutaneous fatty tissue.

To evaluate the degree of agreement between the different measurements methods we reported in this manuscript, we show a comparison of OS, OCT, MRT2* and DSC from an abdominal human sub-cutaneous fatty tissue in Fig. 6. All the measurements consistently show a cooling phase transition in this sample occurs around 9 degrees C, as indicated by the arrows.

**Figure 7:**
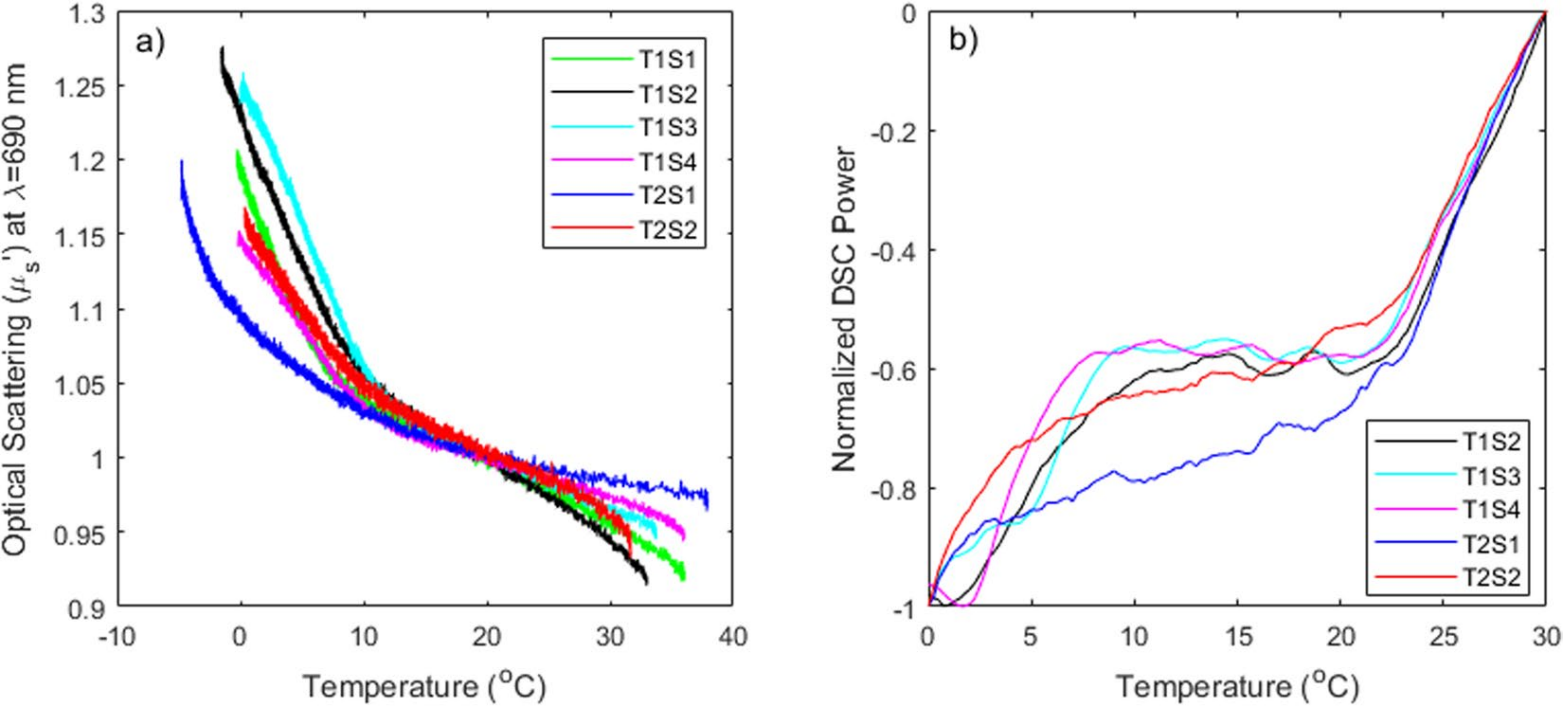
Comparison of scattering change normalized to the scattering coefficient at 20 deg. C (**a**) and DSC Power normalized to the full range of variation in each sample (**b**) across all samples measured (denoted as TnSx, where n is the tissue ID, while x is the sub-sample from that tissue). Note that DSC data was not available for the first sub-sample from the first tissue.

Figure 7 displays a comparison of the normalized scattering changes (panel a) and normalized DSC power across all the samples we have measured. The normalization is necessary to compare samples with different baseline scattering coefficients and with different thermal masses, respectively. We note that all sub-samples from the first tissue behave similarly, both with regards to optical scattering and DSC specific heat, with some degree of variation as expected from a biological tissue. The second sub-samples of the second tissue also displays an apparent transition around 10 degrees, also seen in its DSC data, while the first subsample of the first tissue appears to have a distinct behavior with the acceleration in the scattering increase and higher DSC power draw only showing an inflection closer to 4–5 degrees. Again, it is expected that tissue is not uniform, and it is encouraging to note that this sub-sample diverges with respect to both optical scattering and specific heat behavior, and these two techniques remain in fairly good agreement.

**Figure 8:**
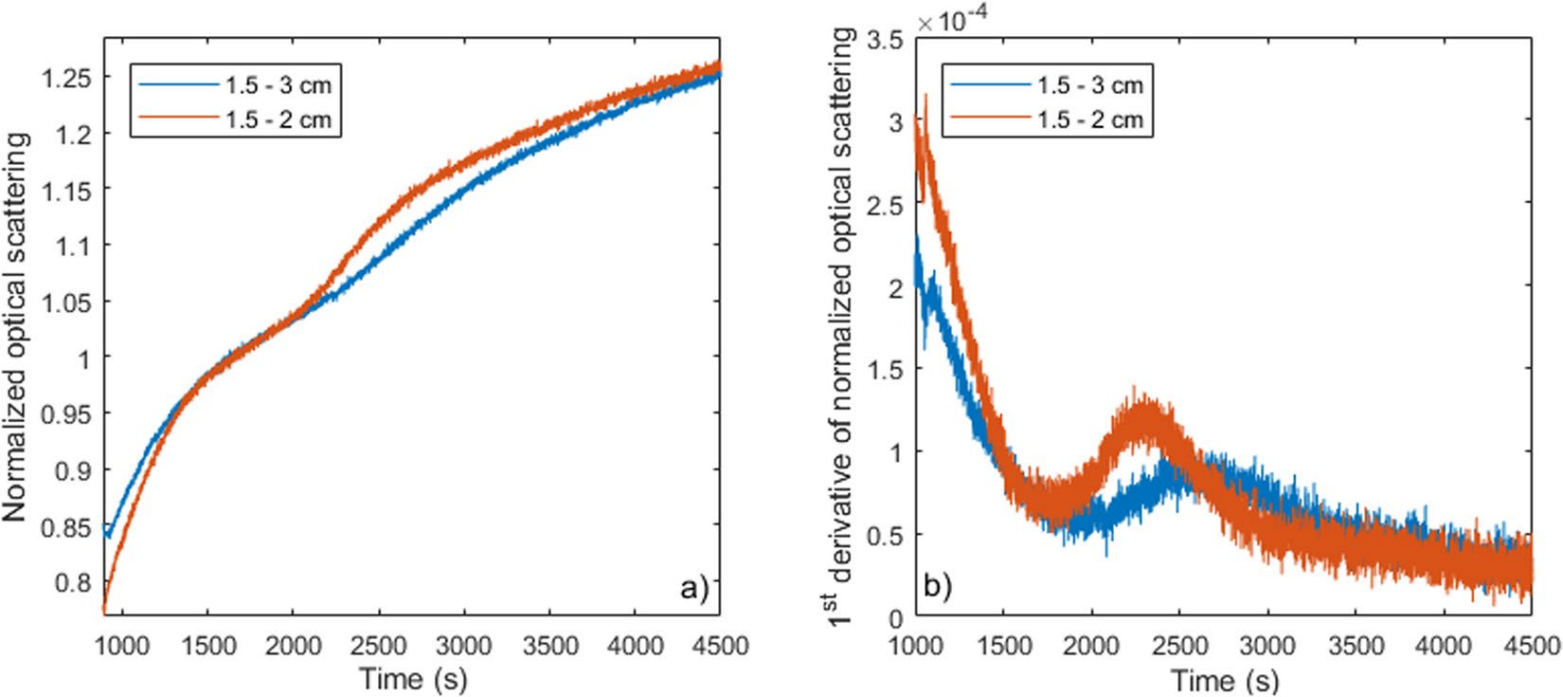
Comparing the scattering at different depths. (**a**) The first two source-detector pairs correspond to a more superficial sensitivity region while using all four optode pairs offers a deeper sensitivity region, where the cooling front arrives later. (**b**) The first derivative of the scattering can show the time of the deflection in the scattering and thus the onset of the phase transition.

To quantify the onset of phase transition at different depths, we compared the average OS computed from the first two source-detector distances (1–1.5 cm) and compared it to the OS time course from the 4 distance data (1–2.5 cm separation), respectively. These are expected to be representative of tissue 4–7 mm under the probe and 8–12 mm under the probe, respectively. Figure 8(a) shows the OS plots at these two depths, while Fig. 8(b) shows the first derivative of the smoothed OS data to help identify changes in the relative change relationship of OS and temperature. The peak of the first derivative of the OS plots likely shows the middle of the phase transition region. The OS of the fat at 1–1.5 cm separation shows earlier transition compared to the deeper location (corresponding to 1–2.5 cm) in the tissue as expected from the cooling front progression from skin to the deeper fatty layer. The first derivative of the OS over time can show the onset of the phase transition and when different optode sets are chosen, the phase transition at different depths can be monitored as shown in Fig. 8(b). Beyond this simple analysis, layered models for light transport could be employed for potentially finer spatial resolution.

## Discussion

Overall, the results from both donor tissues show a remarkable level of agreement between optical scattering measurements and the gold-standard technique for monitoring phase transition processes, differential scanning calorimetry. Additionally, the T2* MR relaxation time and the OCT signal intensity and attenuation measured on one of the samples further demonstrate inflexions in their relationship with temperature around 8–10 degrees during cooling and 13–15 degrees during heating. The exact transition points vary for a number of reasons, for example the slight mis-alignments between the location of the thermal probes, the NIRS sensitivity profile and the MR region of interest, as well as due to sample spatial variation that impacts the behavior of any microscopic piece of tissue harvested vs the sample average. Of note, a potential limitation of our study was that the tissue samples were stored either at 4 °C over night or at −20 °C for several days before we were able to use them in our experiments. While the cold storage may have introduced additional variability in the *ex-vivo* tissue behavior, the repeatability of our data across multiple samples suggests that there were no significant effects of the refrigeration/freezing on the phase transition monitoring measurements.

Another significant agreement is seen in the fact that all methods report a change in the sample intrinsic properties at a temperature several degrees higher during heating than during cooling. This hysteresis was observed during multiple heating/cooling cycles in several samples. This could be due to formation of different polymorphs^13^ of the fat crystals between cooling and heating cycles^19^.

These inflexions in the intrinsic property vs temperature relationship seen across imaging methods strongly suggest an internal change in the structure of the sample. It is in fact visible in OCT data that the cell interior becomes more scattering (opaque) below the transition temperature. This likely is seen macroscopically as a change in the optical scattering coefficient measured with NIRS. The T2* transverse relaxation time depends on both the local microenvironment and molecular level interactions, again suggesting a change in the internal structure of the tissue.

The transition points are likely related to the composition of the sub-cutaneous fat (e.g. saturated vs. unsaturated), and the variability indicates that monitoring may be needed to achieve expected results during cryoprocedures.

## Conclusion

Our results show that the effects of phase change in subcutaneous fat can be detected using NIRS, MR and OCT monitoring. While MR can offer a wealth of information, and OCT is highly useful to understand microscopic tissue changes, perhaps the NIRS monitoring has the most potential to impact clinical cryoprocedures. NIRS measurements could be incorporated into treatment devices inexpensively and, by correlating optical scattering vs. temperature changes, the phase transition point could be predicted. This transition point is likely to occur at somewhat different temperatures from subject to subject due to variation in the amount of saturated vs. unsaturated fat, and potentially other tissue characteristics. NIRS could thus be used to non-invasively monitor fat phase transition *in vivo* to optimize cryosurgery and cryolipolysis. Potentially, this technique can be further utilized to monitor phase change in other surgical procedures such as those using high intensity focused ultrasound, shock waves, radio frequency ablation, IR radiation and laser ablation.
